# Functional TRPV2 and TRPV4 channels in human cardiac c‐kit^+^ progenitor cells

**DOI:** 10.1111/jcmm.12800

**Published:** 2016-02-10

**Authors:** Hui Che, Guo‐Sheng Xiao, Hai‐Ying Sun, Yan Wang, Gui‐Rong Li

**Affiliations:** ^1^ Department of MedicineLi Ka Shing Faculty of MedicineUniversity of Hong KongHong KongChina; ^2^ Xiamen Cardiovascular HospitalMedical College of Xiamen UniversityXiamenFujianChina

**Keywords:** human cardiac c‐kit^+^ progenitor cells, transient receptor potential vanilloid channels, shRNA, proliferation, migration

## Abstract

The cellular physiology and biology of human cardiac c‐kit^+^ progenitor cells has not been extensively characterized and remains an area of active research. This study investigates the functional expression of transient receptor potential vanilloid (TRPV) and possible roles for this ion channel in regulating proliferation and migration of human cardiac c‐kit^+^ progenitor cells. We found that genes coding for TRPV2 and TRPV4 channels and their proteins are significantly expressed in human c‐kit^+^ cardiac stem cells. Probenecid, an activator of TRPV2, induced an increase in intracellular Ca^2+^ (Ca^2+^_i_), an effect that may be attenuated or abolished by the TRPV2 blocker ruthenium red. The TRPV4 channel activator 4α‐phorbol 12‐13‐dicaprinate induced Ca^2+^_i_ oscillations, which can be inhibited by the TRPV4 blocker RN‐1734. The alteration of Ca^2+^_i_ by probenecid or 4α‐phorbol 12‐13‐dicprinate was dramatically inhibited in cells infected with TRPV2 short hairpin RNA (shRNA) or TRPV4 shRNA. Silencing TRPV2, but not TRPV4, significantly reduced cell proliferation by arresting cells at the G0/G1 boundary of the cell cycle. Cell migration was reduced by silencing TRPV2 or TRPV4. Western blot revealed that silencing TRPV2 decreased expression of cyclin D1, cyclin E, pERK1/2 and pAkt, whereas silencing TRPV4 only reduced pAkt expression. Our results demonstrate for the first time that functional TRPV2 and TRPV4 channels are abundantly expressed in human cardiac c‐kit^+^ progenitor cells. TRPV2 channels, but not TRPV4 channels, participate in regulating cell cycle progression; moreover, both TRPV2 and TRPV4 are involved in migration of human cardiac c‐kit^+^ progenitor cells.

## Introduction

It has been demonstrated that several types of tissue‐specific cardiac stem or progenitor cells (expressing specific markers, for example, Sca‐1, c‐kit, *etc*.) are present in adult mammalian hearts including humans. These cells have stem cell‐like capability of self‐renewal and are clonogenic and multipotent 1. Cardiac c‐kit^+^ progenitor cells are a type of cardiac progenitor cells which express the surface tyrosine‐protein kinase c‐Kit (or CD117) 2. Cardiac progenitor cells are believed to be responsible for the renewal of major cardiac cell lineages such as myocytes, endothelial cells, and vascular smooth muscle cells 1, 3. Transplantation of cardiac c‐kit^+^ cells was found to improve left ventricular function in rat and dog myocardial infarction models 3, 4. However, cellular physiology and biology of human cardiac c‐kit^+^ progenitor cells are not yet fully understood.

Our recent studies have demonstrated that several ion channel currents, for example*,* a large conductance Ca^2+^‐activated potassium current (BK_Ca_), a voltage‐gated tetrodotoxin‐sensitive sodium current (I_Na.TTX_) and an inwardly rectifying potassium current (I_Kir_), were heterogeneously expressed in most human cardiac c‐kit^+^ progenitor cells 5, and BK_Ca_, but not I_Na.TTX_ or I_Kir_, regulated cell proliferation. Ca^2+^‐activated potassium current inhibition decreased, and I_Kir_ inhibition increased cell mobility, whereas I_Na.TTX_ suppression had no effect on cell mobility 6. Transient receptor potential channels contain seven subfamilies including TRPC (canonical), TRPM (melastatin), TRPV (vanilloid), TRPA (ankyrin), TRPP (polycystin), TRPML (mucolipin) and TRPN (NOMPC‐like); they are widely described in different mammalian cells 7, 8. Transient receptor potential channels are believed to play important roles in maintaining many physiological and biological homoeostasis 9 as well as regulating cell proliferation, migration, differentiation and pathological processes 10. However, little information is available in literature regarding TRP channels in human cardiac c‐kit^+^ progenitor cells. The present study was designed to investigate the functional expression of TRPV channels and their potential roles in regulating cell proliferation and migration of human cardiac c‐kit^+^ progenitor cells using confocal microscopy, RT‐PCR, Western blot, cell proliferation and migration assays.

## Materials and Methods

### Cell culture

Human cardiac c‐kit^+^ cells were isolated from atrial specimens obtained from coronary artery bypass surgery. Tissue collection was approved by the Ethics Committee of the University of Hong Kong based on the patients’ written consent. The cell isolation, culture and purification were performed following a modified procedure as described previously 5, 11. Our recent study demonstrated that the purified c‐kit^+^ cells were mononuclei cells expressing the stem cell markers CD29 and CD105 in >99% cells, as well as the adult somatic cell marker CD8A in a very limited population of cells (<10%); the cells do not express the hematopoietic stem cell markers CD34 or CD45 5. These characterizations are consistent with the previous reports by other research groups 1, 11.

### Solutions and reagents

Tyrode's solution contains (in mM): 140 NaCl, 5 KCl, 1.0 MgCl_2_, 1.8 CaCl_2_, 10 HEPES, 10 glucose, pH was adjusted to 7.3 using NaOH. All chemicals and reagents were purchased from Sigma‐Aldrich Chemicals (St Louis, MO, USA) unless otherwise specified. 4α‐phorbol 12, 13‐didecanoate (4α‐PDD) was purchased from Calbiochem, (San Diego, CA, USA). Stock solutions were dissolved in dimethyl sulfoxide and divided into aliquots and stored at −20°C.

### Reverse transcript‐polymerase chain reaction

The reverse transcript‐polymerase chain reaction (RT‐PCR) was performed with a procedure described previously 12, 13. Briefly, total RNA was isolated using the TRIzol method (Invitrogen) from human cardiac c‐kit^+^ progenitor cells and then treated with DNase I (Invitrogen). Reverse transcription (RT) was performed with RT system (Promega, Madison, WI, USA) protocol in 20 μl reaction mixture. RNA (1 μg) was used in the reaction, and a combination of oligo (dT) and random hexamer primers was used for the initiation of cDNA synthesis. After RT, the reaction mixture (cDNA) was used for polymerase chain reaction (PCR). The forward and reverse PCR oligonucleotide primers chosen to amplify the cDNA are listed in Table 1. PCR was performed by a Promega PCR system with Taq polymerase and accompanying buffers. The cDNA in 2 μl aliquots was amplified by a DNA thermal cycler (MyCycler; Bio‐Rad, Hercules, CA, USA) in a 25 μl reaction mixture as described previously 12, 13. The PCR products were electrophoresed through a 1.5% agarose gel, and the amplified cDNA bands were visualized by ethidium bromide staining. The bands imaged by Chemi‐Genius Bio Imaging System (Syngene, Cambridge, UK).

**Table 1:** Human gene‐specific primers for RT‐PCR

Gene (accession no.)	Primer sequences(5′–3′)	Fragment size (bp)
TRPV1 (NM_080706)	Forward: GCCGTTTCATGTTTGTCTA Reverse: GAGCAGGAGGATGTAGGTG	296
TRPV2 (NM_016113)	Forward: TGTAGCCCTGGTGAGCCT Reverse: CCAACGGTCAGCATCACA	423
TRPV3 (NM_145068)	Forward: GCGTGGAGGAGTTGGTAG Reverse: GGCGTCTCACCGAAGTAG	403
TRPV4 (NM_001177431)	Forward: TGGCTTCTCGCATACCGT Reverse: GGCTCTGGCGTTGGCTTA	431
TRPV5 (NM_019841)	Forward: CACCTGCCAACTACGACG Reverse: TTCCGCTCCAGCATCACT	193
TRPV6 (NM_018646)	Forward: GCTTTGCTTCAGCCTTCT Reverse: CAGTGAGTGTCGCCCATC	241
GAPDH (J02642)	Forward: AACAGCGACACCCACTCCTC Reverse: GGAGGGGAGATTCAGTGTGGT	258

TRPV, transient receptor potential vanilloid; GAPDH, glyceraldehyde 3‐phosphate dehydrogenase.

### Western blotting analysis

Western blotting analysis was conducted to determine the related proteins with the procedures as described previously 13, 14. Briefly, human cardiac c‐kit^+^ progenitor cells were lysed with a modified RIPA buffer, and cell lysates were then centrifuged at 12,000 g for 15 min. at 4°C. After transferring the supernatant to a fresh ice‐cold tube, protein concentration was determined with Bio‐Rad protein assay. Equal concentrations of proteins were mixed with SDS sample buffer and denatured at 95°C for 5 min. The samples were resolved with 8% SDS–page gels which were then transferred onto nitrocellulose membranes. The membranes were blocked with 5% non‐fat dried milk in TTBS (0.1% Tween‐20) for 1 hr, and then probed with primary antibody (1:1000–2000) overnight at 4°C. Anti‐TRPV2, anti‐cyclin D and anti‐cyclin E antibodies were from Santa Cruz Biotechnology (Santa Cruz, CA, USA); anti‐TRPV4 was from Alomone (Jerusalem, Israel); anti‐ERK1/2, anti‐pERK1/2, anti‐Akt, anti‐pAkt were from Cell Signaling (Danvers, MA, USA). After washing with TTBS, the membranes were incubated with goat antimouse IgG‐horseradish peroxidase (HRP) at 1:4000 dilution in TTBS at room temperature for 1 hr. Membranes were washed again with TTBS then processed on x‐ray films using an enhanced chemiluminescence detection system (ECL; GE Healthcare, Bio‐Science AB, Uppsala, Sweden). The relative band intensities were measured by image analysis software Gel‐Pro Analyser (Media Cybernetics, Inc., Rockville, MD, USA).

### Intracellular Ca^2+^ measurements

Intracellular Ca^2+^ (Ca^2+^_i_) activity was measured in cultured human cardiac c‐kit^+^ progenitor cells using fluo3‐AM (Biotium, Hayward, CA, USA) as described previously 12, 13. Briefly, the cells were loaded with 5 μM Fluo3‐AM for 30 min. at 37°C, and then incubated in Tyrode's solution for 1 hr. Ca^2+^_i_ was determined by exciting Fluo‐3 with a 488 nm argon‐ion laser and detected emission at 506 nm in human Tyrode's solution. Ca^2+^_i_ activity was monitored every 10 sec. using confocal microscopy (Olympus FV300, Tokyo, Japan) at room temperature (23–24°C).

### Lentiviral shRNA construction and cell transfection

The optimal 21‐Mer targets in target gene were selected according to the recommendation of Open Biosystems (https://www.openbiosystems.com/) as described previously 14. Three optimal 21‐mer short hairpin RNAs (shRNAs) targeting human TRPV2 gene are CCTAGTGATGATCTCGGACAA, CTTCTTAAACTTCCTGTGTAA and CCTTCTGATCTACTTAGTCTT; three optimal 21‐mer shRNAs targeting human TRPV4 gene are GCCAGTGTATTCCTCGCTTTA, GCCAACATGAAGGTGTGCAAT and CGCTGCAAACACTACGTGGAA. Oligos were then cloned into pLKO.1 by following the Addgene (http://www.addgene.org/tools/protocols/plko/) protocol. Lentiviral shRNA targeting eGFP was used as a control to determine the knockdown efficiency using fluorescence microscopy. The scramble shRNA lentiviral particles containing a scrambled shRNA sequence that will not lead to the specific degradation of any cellular mRNA was used as a negative control for target shRNA lentiviral particles.

Lentiviral transduction was performed in HEK‐293T cells *via* transient cotransfection involving a three plasmid expression system as described 15. Human cardiac c‐kit^+^ progenitor cells for infection were plated in 6‐well plates, 1 × 10^6^ cells/well. After 24 hrs, 100 μl pools of shRNAs were added to the cells in fresh medium containing 8 μg/ml polybrene. 48 hrs later, cells were selected in fresh medium containing puromycin (3 μg/ml); the selected cells were then cultured for 3–5 days. The puromycin‐resistant cells were pooled and the knockdown efficiency was verified by both RT‐PCR and Western blot analysis, the most effective shRNA was chosen for the further experimental study.

### Cell proliferation assays

3‐(4,5‐dimethyl‐thiazol‐2‐yl)‐2,5‐diphenyl tetrazolium bromide (MTT) and [^3^H]‐thymidine incorporation methods were employed as described previously to determine the effects of silencing TRP channels with specific shRNAs on cell proliferation 14, 16. For MTT assay, human cardiac c‐kit^+^ progenitor cells were plated into 96‐well plate at a density of ~5000 cells/well in 200 μl complete culture medium. After 8 hrs recovery, the cells were incubated in culture medium containing lentiviral particles for 24 hrs, the medium was then replaced with normal culture medium. Following 72 hrs incubation, 20 μl PBS‐buffered MTT (5 mg/ml) solution was added to each well and the plates were incubated at 37°C for an additional 4 hrs. The medium was removed and 100 μl dimethyl sulfoxide was added to each well to dissolve the purple formazan crystals. The plates were read (wavelengths: test, 570; reference, 630 nm) using a Quant microplate spectrophotometer (Bio‐Tek Instruments, Inc., Winooski, VT, USA). Results were standardized using control group values.

For [^3^H]‐thymidine incorporation method, human cardiac c‐kit^+^ progenitor cells were plated into 96‐well plate at a density of ~5000 cells/well in 200 μl complete culture medium for 8 hrs, the culture medium was replaced with the medium containing lentiviral particles and incubated for 24 hrs. Then the medium was changed to normal culture medium. Following 48 hrs incubation, 1 μCi (0.037 MBq) [^3^H]‐thymidine (GE Healthcare) was added into each well for 24 hrs. The cells were then harvested and transferred to a nitrocellulose coated 96‐well plate *via* suction. The nitrocellulose membrane was washed with water flow and the plate was air‐dried at 50°C overnight. Liquid scintillation cocktail (20 μl per well) was then added to each well. Counts per minute for each well were read by a TopCount microplate scintillation and luminescence counter (PerkinElmer, Waltharn, MA, USA).

### Flow cytometric analysis

Cell cycle distribution of human cardiac c‐kit^+^ progenitor cells was determined by flow cytometry (FC500, Beckman, Fullerton, CA, USA) as described previously 14. Briefly, cells were lifted with 0.25% trypsin, washed with PBS and fixed with ice‐cold 70% ethanol. After removal of ethanol by centrifugation, the cell pellets were washed with PBS. Then cells were incubated with propidium iodide/PBS staining buffer (propidium iodide: 20 mg/ml, RNaseA100 mg/ml and 0.1% Triton‐X 100) at 37°C for 30 min. Data were acquired with CellQuest software (BD Biosciences, San Jose, CA, USA), and the percentages of G0/G1 boundary, S and G2/M phase cells were calculated with MODFIT LT software (BD Biosciences).

### Cell mobility assays

The cell mobility was determined with wound healing and transwell assays in human cardiac c‐kit^+^ cells as described previously 16, 17. Briefly, a standard wound was created by scratching the cell monolayer with a sterile 200‐μl plastic pipette tip and line makers were made at the bottom of plates to indicate the wound edges. After removing cell fragments, the cells were incubated at 37°C with medium containing 1% FBS (fetal bovine serum, to limit cell proliferation) for 8 hrs. Then the defined areas of the wound gap were photographed under a phase contrast microscope (Olympus, Tokyo, Japan). The migrated cells on the images were counted to assess cell mobility under different conditions. Experiments were performed in triplicate.

Transwell assay was performed with a modified Boyden chamber with 8‐μm‐pore polycarbonate membranes (Corning Inc., Corning, NY, USA) as described previously 16, 17. The chambers were pre‐coated with 700 μl serum‐free medium for at least 1 hr. After the pre‐coated medium was removed, ~5000 viable human cardiac c‐kit^+^ cells were plated into the upper chamber in 200 μl medium containing 1% FBS, and the lower chamber containing 600 μl medium containing 1% FBS. The plates were incubated at 37°C in 5% CO_2_ for 8 hrs, they were then washed with PBS for three times, fixed with formaldehyde for 15 min. at room temperature and stained with crystal violet for 15 min. After washing with PBS to thoroughly remove the dye, non‐migrated cells on the upper surface of the membrane were scraped off with cotton swabs. The migrated cells on the lower surface of the membrane were counted in five representative fields under a microscope. Experiments were performed in triplicate.

### Statistical analysis

Group data were expressed as mean ± S.E.M. Paired and/or unpaired Student's *t*‐test were used as appropriate to evaluate the statistical significance of differences between two group means, and anova was used for multiple groups. Values of *P* < 0.05 were considered to be statistically significant.

## Results

### Gene and protein expression of TRPV channels in human cardiac c‐kit^+^ progenitor cells

**Figure 1:**
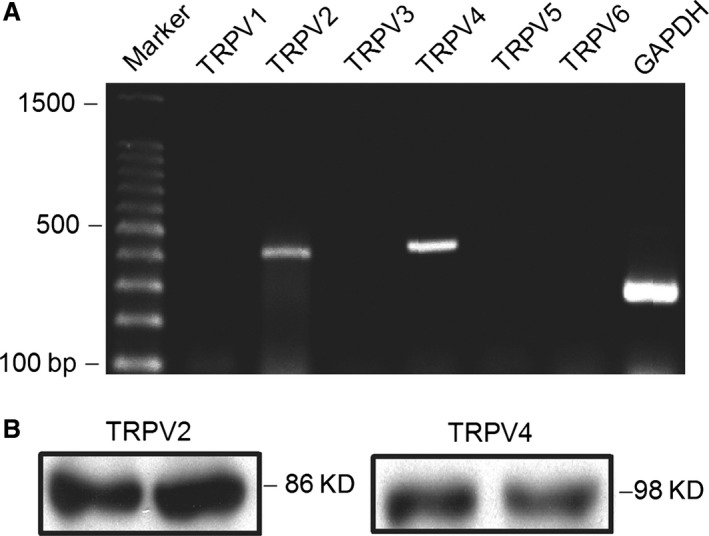
Gene and protein expression of TRPV channels in human cardiac c‐kit^+^ progenitor cells. (**A**) Images of RT‐PCR of TRPV channels expression. (**B**) Western blots of TRPV2 and TRPV4 channels in human cardiac c‐kit^+^ progenitor cells. TRPV, transient receptor potential vanilloid.

Figure 1A shows the images of PCR products for mRNA expression of TRPV genes. Transient receptor potential vanilloid 2 and TRPV4 were abundant in human cardiac c‐kit^+^ progenitor cells. Figure 1B displays the Western blots for protein expression of TRPV2 and TRPV4 channels. These data suggest that TRPV2 and TRPV4 channels are present in human cardiac c‐kit^+^ progenitor cells.

### Functional TRPV2 and TRPV4 channels mediate intracellular Ca^2+^ activity in human cardiac c‐kit^+^ progenitor cells

**Figure 2:**
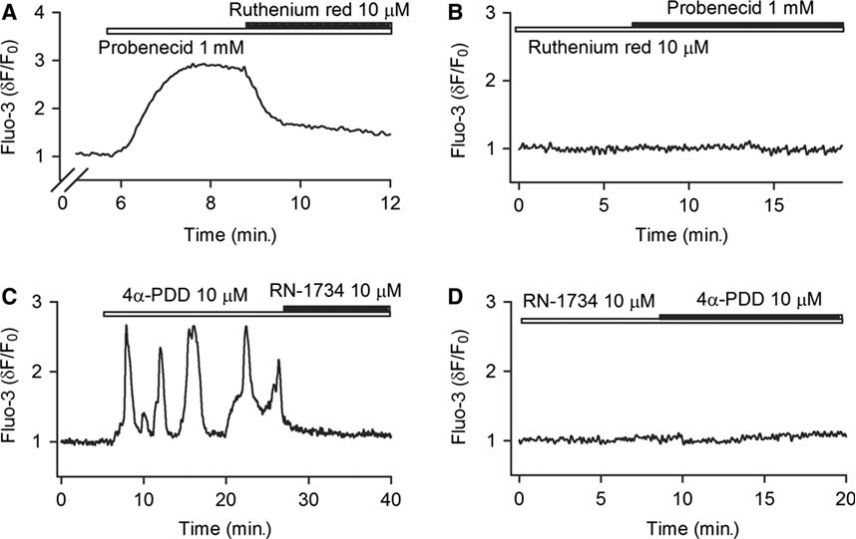
Ca^2+^ signals mediated by TRPV2 and TRPV4 channels in human cardiac c‐kit^+^ progenitor cells. (**A**) TRPV2 channel activator (probenecid, 1 mM) induced a long‐lasting Ca^2+^_i_ increase, the effect was countered by the TRPV inhibitor ruthenium red (10 μM). (**B**) Ruthenium red pre‐treatment prevented the Ca^2+^_i_ increase induced by probenecid. (**C**) Ca^2+^_i_ oscillations induced by TRPV4 channel activator (4α‐PDD, 10 μM) was decreased by the TRPV4 inhibitor RN‐1734 (10 μM). (**D**) RN‐1734 pre‐treatment prevented the Ca^2+^_i_ oscillations induced by 4α‐PDD (*n* = 30 for each treatment). TRPV, transient receptor potential vanilloid.

Transient receptor potential vanilloid 2 or TRPV4 channels mediates Ca^2+^ entry and are activated by the specific agonist probenecid 18 or 4‐alpha‐phrobol 12,13‐didecanoate (4α‐PDD) 19 respectively. We therefore used the TRPV2 activator probenecid and TRPV4 activator 4α‐PDD to determine whether TRPV2 or TRPV4 could enhance Ca^2+^ signals in human cardiac c‐kit^+^ progenitor cells. Figure 2A shows that probenecid at 1 mM cause a long‐lasting Ca^2+^_i_ increase, which was reversed by co‐application of the TRPV2 channel blocker ruthenium red (10 μM, *n* = 30). Pre‐incubation with ruthenium red fully prevented Ca^2+^_i_ increase by probenecid (Fig. 2B, *n* = 31). These results indicate that functional TRPV2 channels are present and mediate Ca^2+^_i_ increase in human cardiac c‐kit^+^ progenitor cells.

Figure 2C displays that the TRPV4 channel activator 4α‐PDD (10 μM) induced Ca^2+^_i_ oscillations, and the effect was suppressed by the TRPV4 channel inhibitor RN‐1734 (10 μM). Cell population with Ca^2+^_i_ oscillations was increased to 66.7% (30 of 45, *P* < 0.05 *versus* control, 22.2% in 10 of 45) of cells with 4α‐PDD. No Ca^2+^ oscillations were observed with 4α‐PDD in cells pre‐treated with RN‐1734 (Fig. 2D, *n* = 30). These results indicate that functional TRPV4 channels are present in human cardiac c‐kit^+^ progenitor cells and mediate Ca^2+^_i_ oscillations.

### Silencing TRPV2 and TRPV4 channels

**Figure 3:**
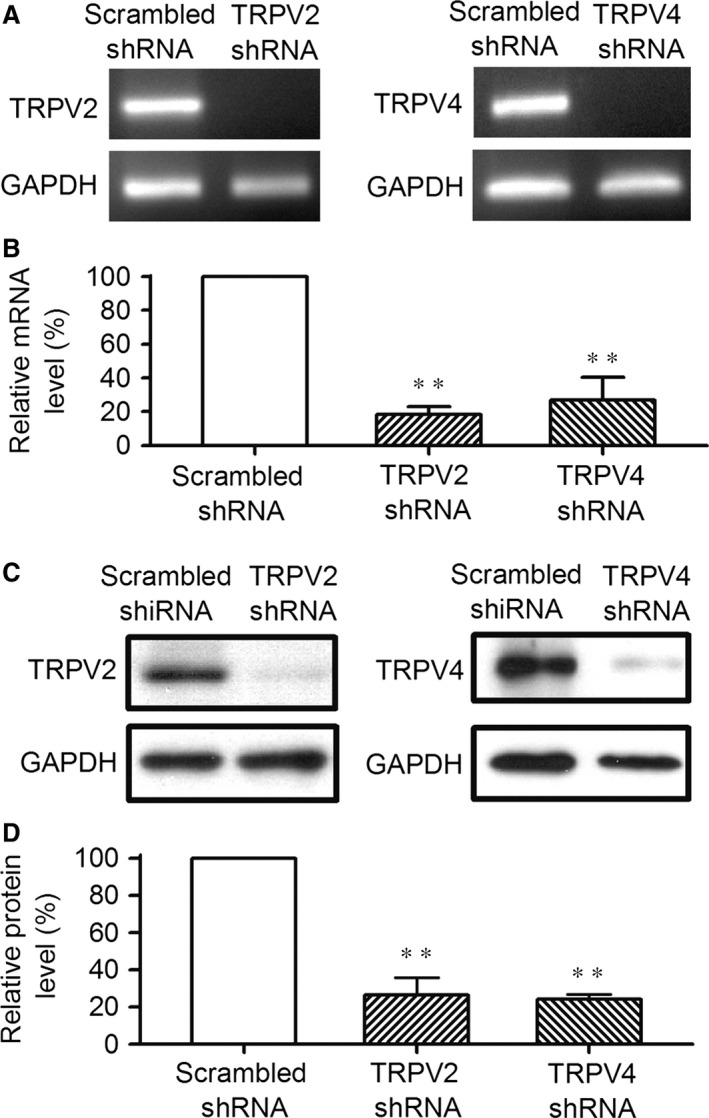
Silencing TRPV2 and TRPV4 channels with corresponding shRNA. (**A**) Images of RT‐PCR in cells infected by scrambled shRNA, TRPV2 shRNA or TRPV4 shRNA for 96 hrs in human cardiac c‐kit^+^ progenitor cells. (**B**) Relative mean values of mRNA for TRPV2 and TRPV4 in cells infected by corresponding specific shRNAs (*n* = 3, **P < 0.01 *versus* scrambled shRNA). (**C**) Western blots of cells infected by scrambled shRNA, TRPV2 shRNA or TRPV4 shRNA. (**D**) Relative mean values of TRPV2 and TRPV4 protein in cells infected with corresponding shRNAs (*n* = 3, **P < 0.01 *versus* scrambled shRNA). TRPV, transient receptor potential vanilloid.

To study whether TRPV2 and TRPV4 channels are involved in regulating cell proliferation and migration in human cardiac c‐kit^+^ progenitor cells, shRNA targeting to TRPV2 or TRPV4 gene was used to silence TRPV2 or TRPV4 channels. Figure 3 displays that the mRNAs and proteins of TRPV2 or TRPV4 were remarkably reduced in human cardiac c‐kit^+^ progenitor cells infected with TRPV2 shRNA or TRPV4 shRNA (Fig. 3A and C). The mean values of gene and protein expression of these channels were significantly reduced in cells infected by TRPV2 shRNA or TRPV4 shRNA (Fig. 3B and D, *n* = 3, *P* < 0.01 *versus* scrambled shRNA).

**Figure 4:**
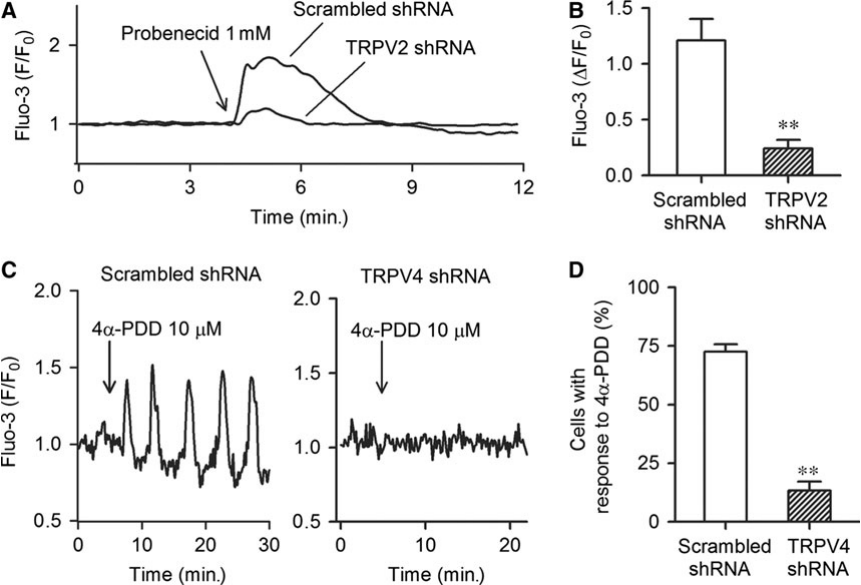
Functional activity of TRP channels in cells infected by corresponding shRNAs. (**A**) Probenecid‐induced Ca^2+^_i_ increase in cells infected by TRPV2 shRNA or scrambled shRNA. (**B**) Mean values of Ca^2+^_i_ increase induced by the TRPV2 activator probenecid (1 mM) in cells infected by TRPV2 shRNA (n = 45) or scrambled shRNA (*n* = 42, **P < 0.01 *versus* scrambled shRNA). (**C**) 4α‐PDD‐induced Ca^2+^_i_ oscillations in cells infected by scrambled shRNA or TRPV4 shRNA. (**D**) Mean percentage of cells with Ca^2+^_i_ oscillations in the presence of the TRPV4 activator 4α‐PDD (10 μM) (*n* = 62, **P < 0.01 *versus* scrambled shRNA, n = 45). TRPV, transient receptor potential vanilloid.

To further examine the effect of silencing TRPV2 and TRPV4 channels, Ca^2+^_i_ activity was determined in cells infected by the corresponding shRNAs. Figure 4A shows that Ca^2+^_i_ is increased by the TRPV2 activator probenecid (1 mM) in cells infected by TRPV2 shRNA or scrambled shRNA. The Ca^2+^_i_ increase by probenecid was remarkably reduced in cells infected by TRPV2 shRNA compared with cells infected by scrambled shRNA. The mean value (Fig. 4B) of relative Ca^2+^_i_ increase level by probenecid was 1.21 ± 0.19 in cells infected by scrambled shRNA (*n* = 45), while was decreased to 0.24 ± 0.08 in cells infected by TRPV2 shRNA (*n* = 42, *P* < 0.01 *versus* scrambled shRNA).

Figure 4C shows the Ca^2+^_i_ oscillations induced by the TRPV4 channel activator 4α‐PDD (10 μM) in cells infected by TRPV4 shRNA or scrambled shRNA. Ca^2+^_i_ oscillations induced by 4α‐PDD were significant in cells infected by scrambled shRNA, but not by TRPV4 shRNA. The mean percentage (Fig. 4D) of the cell number with positive response to 4α‐PDD was 72.6 ± 3.0% in cells infected by scrambled shRNA (*n* = 45) compared to 13.3 ± 3.8% in cells infected by TRPV4 shRNA (*n* = 62, *P* < 0.01 *versus* scrambled shRNA).

### TRPV channels and cell proliferation in human cardiac c‐kit^+^ progenitor cells

**Figure 5:**
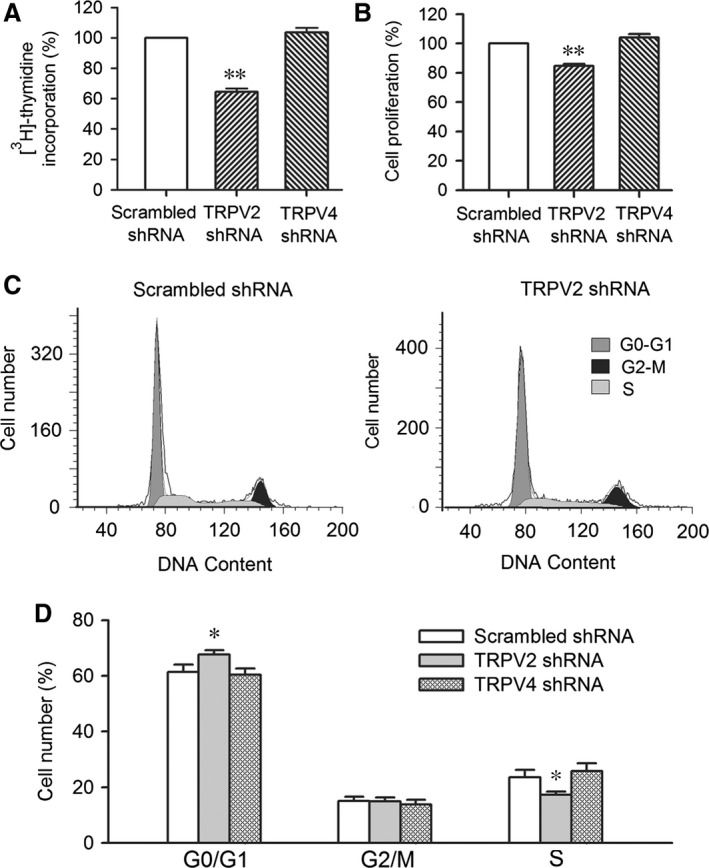
Effect of silencing TRPV2 or TRPV4 channels on cell proliferation. (**A**) Mean values of [^3^H]‐thymidine incorporation in cells infected by TRPV2 shRNA, or TRPV4 shRNA (*n* = 3, **P < 0.01 *versus* scrambled shRNA). (**B**) Cell proliferation in cells infected by TRPV2 shRNA or TRPV4 shRNA (*n* = 3, **P < 0.01 *versus* scrambled shRNA). (**C**) Flow cytometry graphs in cells infected by scrambled shRNA or TRPV2 shRNA. (**D**) Mean percentage values of cell cycling population at different phases in cells infected by corresponding shRNAs (*n* = 3, *P < 0.05 *versus* scrambled shRNA). TRPV, transient receptor potential vanilloid.

We determined how the cell proliferation and cell cycle were affected in cells infected by TRPV2 shRNA or TRPV4 shRNA using [^3^H]‐thymidine incorporation and MTT assays. Figure 5A illustrates that [^3^H]‐thymidine incorporation rate was significantly reduced by 35.7 ± 2.3% in cells infected by TRPV2 shRNA (*n* = 4, *P* < 0.01 *versus* scrambled shRNA), but not in cells infected by TRPV4 shRNA. Similar results were observed in MTT assay (Fig. 5B). These data suggest that TRPV2, but not TRPV4 channels, participate in regulating cell proliferation.

Cell cycling progression was determined in human cardiac c‐kit^+^ progenitor cells infected with different shRNAs. Figure 5C displays the flow cytometry graphs in cells infected by scrambled shRNA or TRPV2 shRNA. The mean percentage values of cell population at different cycling phases are illustrated in Figure 5D. The cell population at G0/G1 boundary was significantly increased (*n* = 4, *P* < 0.05 *versus* scrambled shRNA), and cell population at S phase was significantly reduced (*P* < 0.05 *versus* scrambled shRNA) in cells infected by TRPV2 shRNA, but not in cells infected by TRPV4 shRNA. These results suggest that TRPV2, but not TRPV4 channels, regulate cell proliferation *via* promoting cell cycling progression to S phase from G0/G1 boundary transition.

### TRPV channels and cell migration in human cardiac c‐kit^+^ progenitor cells

**Figure 6:**
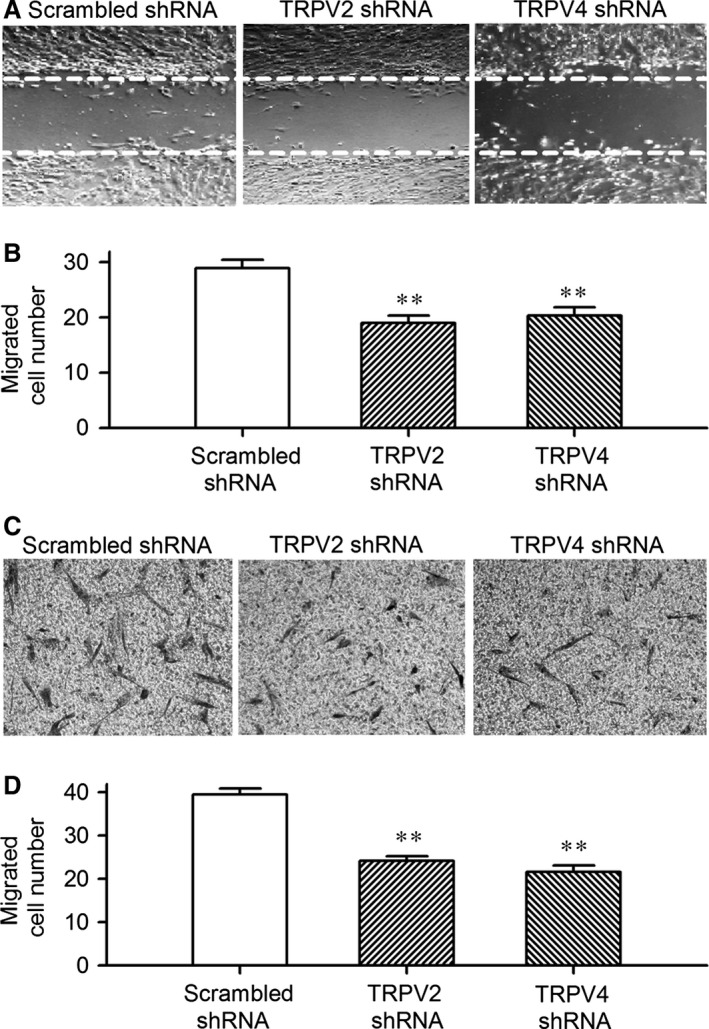
TRPV2 and TRPV4 channels and cell migration. (**A**) Images of wound healing assay with a pipette tip to create acellular areas in cells infected by scrambled shRNA, TRPV2 shRNA or TRPV4 shRNA. (**B**) Mean values of number of migrated human cardiac c‐kit^+^ progenitor cells infected by the corresponding shRNA (*n* = 3, **P < 0.01 *versus* scrambled shRNA). (**C**) Images of human cardiac c‐kit^+^ progenitor cells on the lower surface membrane in Transwell assay. (**D**) Mean values of migrated human cardiac c‐kit^+^ progenitor cells on the lower surface membrane in cells infected by TRPV2 shRNA or TRPV4 shRNA (*n* = 3, **P < 0.01 *versus* scrambled shRNA). TRPV, transient receptor potential vanilloid.

The potential effects of TRPV2 and TRPV4 channels on cell mobility were determined in cells infected with TRPV2 shRNA or TRPV4 shRNA using wound‐healing and transwell assays. In wound‐healing assay, the human cardiac c‐kit^+^ progenitor cell migration into the acellular area was determined after 8‐hrs culture as shown in Figure 6A. Figure 6B illustrates the mean values of cell number migrated into the acellular area. The migrated cell number was significantly reduced in cells infected by TRPV2 shRNA or TRPV4 shRNA (*n* = 4, *P* < 0.01 *versus* scrambled shRNA).

To exclude the potential contamination caused by cell proliferation on cell migration, transwell assay was performed in cells infected by TRPV2 shRNA or TRPV4 shRNA by seeding the cells to the upper chamber of the transwell, and counting the cell number on the lower surface of the membrane after 8 hrs (Fig. 6C). The results obtained in transwell assay was similar to that observed in wound healing assay; the number of the migrated cells was reduced by silencing TRPV2 or TRPV4 gene (Fig. 6D, *n* = 4, *P* < 0.01 *versus* scrambled shRNA). These results suggest that TRPV2 and TRPV4 participate in cell migration in human cardiac c‐kit^+^ progenitor cells.

### TRPV2 and TRPV4 channels and intracellular signals

**Figure 7:**
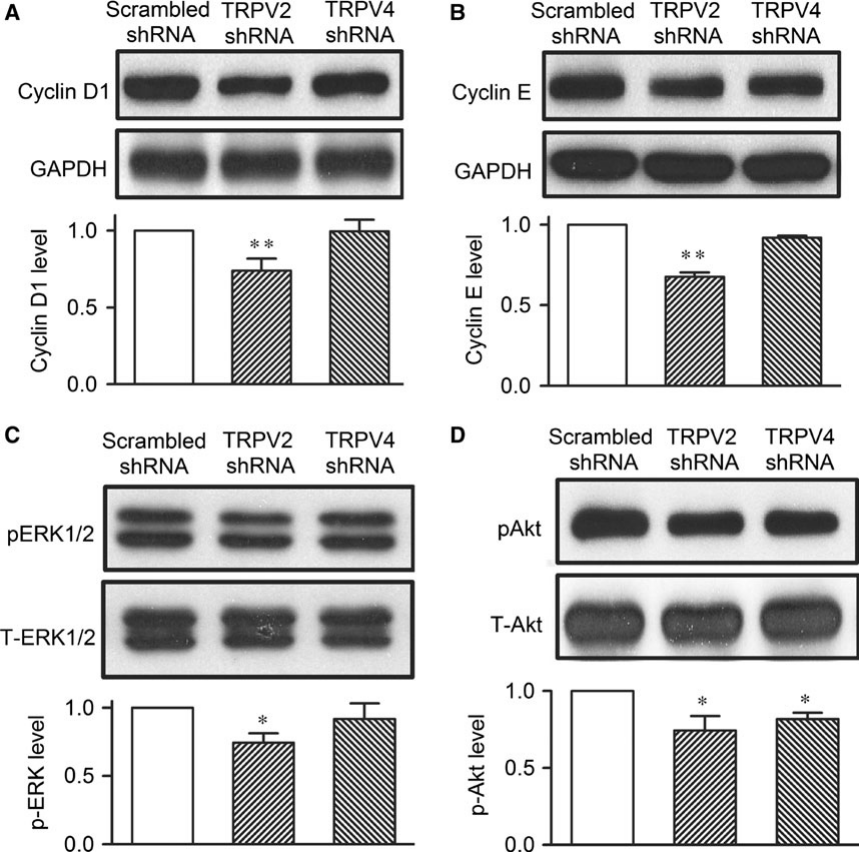
Intracellular signals in human cardiac c‐kit^+^ progenitor cells. (**A**) Expression levels of cyclin D1 in cells infected by scrambled shRNA, TRPV2 shRNA or TRPV4 shRNA (n = 3, **P < 0.01 versus scrambled shRNA). (**B**) Expression levels of cyclin E in cells infected by the corresponding shRNA. (**C**) Expression levels of phosphorylated ERK1/2 in cells infected by the corresponding shRNA. (**D**) Expression level of pAkt in cells infected by the corresponding shRNA (n = 3, *P < 0.05 versus scrambled shRNA). TRPV, transient receptor potential vanilloid.

To investigate the potential molecular pathways in which TRPV2 and TRPV4 modulate cell cycling progression and/or migration, we determined the cell cyclin kinases cyclin D1 and cyclin E1, and the mitogen‐activated protein kinase pERK1/2, and the survival kinase pAkt in human cardiac c‐kit^+^ progenitor cells infected by TRPV2 shRNA or TRPV4 shRNA (Fig. 7). Cyclin D1, cyclin E and pERK1/2 were remarkably decreased in cells with silenced TRPV2 channels (*n* = 4, *P* < 0.05 or *P* < 0.01 *versus* scrambled shRNA), but not with silenced TRPV4 channels. Interestingly, pAkt was reduced in cells infected by TRPV2 shRNA or TRPV4 shRNA (*n* = 4, *P* < 0.05 *versus* scrambled shRNA). These results suggest that TRPV2 channels affect cell proliferation by regulating cyclin D1, cyclin E and pERK1/2; TRPV2 and TRPV4 may contribute to cell migration through pAkt in human cardiac c‐kit^+^ cells.

## Discussion

The TRPV subfamily (vanilloid receptors) comprises channels critically involved in nociception and thermosensing (TRPV1, TRPV2, TRPV3 and TRPV4), whereas TRPV5 and TRPV6 are involved in renal Ca^2+^ absorption/reabsorption 7. The TRPV channels are activated by endogenous ligands, heat, mechanical and osmotic stress by responding to local environmental changes. It has been documented that TRPV channels are widely expressed in vasculature, including smooth muscle cells, endothelial cells, as well as in peri‐vascular nerves 20, 21. The TRPV channels are also sensitive to modulation by exogenous small molecules, for example, that TRPV1 is activated by the food molecule capsaicin 20, 21. This compound has been recently found to bind to TRPV1 and stabilize the open state of the channel 22. An earlier study in isolated working rat heart demonstrated that capsaicin mediated a significant change in cardiac and arterial function (reducing cardiac contractile function and coronary artery flow) *via* activating capsaicin receptors (TRPV1 channels) and therefore increasing endothelin release from sensory nerve terminals 23. The increasing evidence shows that TRPV channels play important roles in cardiovascular physiology and pathophysiology 24, 25.

In this study, we provide the novel information that functional TRPV2 and TRPV4 channels are present in human cardiac c‐kit^+^ progenitor cells. These TRP channels participate in regulating cell proliferation and/or mediates cell migration in human cardiac c‐kit^+^ progenitor cells. The TRP channels are the non‐selective cation channels which are expressed ubiquitously and function as cellular sensors, and participate in various cell functions 7. It has been demonstrated that TRPC1, TRPC4, TRPC6, TRPC7, TRPV2, TRPV4, TRPM4 or TRPM7 are present in the heart. However, information on TRP channels was mainly collected from whole heart tissues 26, 27 or cardiac myocytes 28, 29. In the present study, we provided the novel information that functional TRPV2 and TRPV4 channels are abundantly expressed in human cardiac c‐kit^+^ progenitor cells.

It is generally recognized that TRPV2 is a Ca^2+^‐permeable channel regulated by insulin‐like growth factors 30. It was initially believed that TRPV2 was a thermo‐sensitive channel activated by noxious heat (>53°C) 31. However, this notion has been challenged by a recent report from Park and colleagues 32. They demonstrated that TRPV2 knockout mice showed a similar response to thermal and mechanical nociception to that of wild‐type mice, and concluded that TRPV2 is not essential for heat or mechanical nociception or hypersensitivity in the adult mouse. Transient receptor potential vanilloid 2 is actually expressed broadly in brain, spleen, lung, *etc*. and is involved in regulating macrophage migration, phagocytosis, cytokine release and also in immune response 33, 34, 35. Transient receptor potential vanilloid 2 is also expressed in cardiovascular system 35. Iwata and colleagues first reported cardiac specific overexpression of TRPV2 and demonstrated that blockade of TRPV2 channels prevented ventricular dilation and fibrosis, ameliorated contractile dysfunction in dilated cardiomyopathy in animal models 36. Interestingly, a recent study reported that the TRPV2 activator probenecid mediated a non‐injurious cardiac positive inotropic effect *via* increasing Ca^2+^ release from sarcoplasmic reticulum in a mouse model 18.

In this study, we demonstrated that the TRPV2‐specific activator probenecid induces a long‐lasting Ca^2+^_i_ increase which could be prevented by the blocker ruthenium red. Silencing TRPV2 channels reduced the Ca^2+^_i_ increase by probenecid, and decreased cell proliferation by arresting the cells at G0/G1 boundary *via* inhibiting cyclin D1, cyclin E and phosphorylated ERK1/2, as well as reduced cell migration by inhibiting phosphorylated Akt. These results suggest that TRPV2 channels participate in cell proliferation and migration in human cardiac c‐kit^+^ progenitor cells. These effects are consistent with observations that TRPV2 channels in macrophage cells 33, 35 prostate cancer cells 37, and human preadipocytes 14 are pro‐proliferation and/or pro‐migration.

Transient receptor potential vanilloid 4 is also a type of non‐selective cation channels with considerable Ca^2+^ conductance. TRPV4 channels expressed in HEK 293 cells could be activated by cell swelling, heating or chemical agonists *via* different pathways 38. It has been demonstrated that TRPV4 channels are expressed in different native tissues/cells and serve different cellular functions. In vascular muscles, the influx of extracellular Ca^2+^ mediated by TRPV4 channels may act directly on Ca^2+^ sensitive endothelial cells to release soluble vasodilatory factors or initiate processes that promote dilation through hyperpolarization of the membrane of underlying vascular smooth muscle cells 39. In neuronal cells, TRPV4 channels stimulate Ca^2+^‐induced Ca^2+^ release in astrocytic endfeet and mediate neurovascular coupling responses 40. In adipose tissue TRPV4 channels regulate adipose oxidative metabolism, inflammation and energy homoeostasis 41, and also participate in adipogenesis in human preadipocytes *via* phosphorylating Akt kinase 14. In addition, TRPV4 channels modulate chondrogenic differentiation and maintain bone homoeostasis in bone tissues 42.

In this study, we demonstrated that TRPV4 channels were present in human cardiac c‐kit^+^ progenitor cells, and activation of the channels by the agonist 4α‐PDD initiated spontaneous Ca^2+^_i_ oscillations. Though TRPV4 channels were reported to regulate cell proliferation in human oesophageal epithelial cells 43 and cultured porcine endothelial cells 44, we found that silencing TRPV4 channels had no effect on proliferation of human cardiac c‐kit^+^ progenitor cells in the present study or human preadipocytes in a previous report 14. Interestingly, silencing TRPV4 decreased cell migration in human cardiac c‐kit^+^ progenitor cells. This effect is supported by the observation in rat pulmonary arterial smooth muscle cells, in which activation of TRPV4 channels by 4α‐PDD enhances cell migration without affecting proliferation 45. The reduced pAkt in cells with silenced TRPV4 channels suggests that the cell mobility is regulated *via* pAkt in human cardiac c‐kit^+^ progenitor cells. The limitation of this study was that the number of cases is small and may influence the results due to associated pathologies; nonetheless, this may not affect the conclusion from the present study.

Collectively, in this study we demonstrate for the first time that TRPV2 and TRPV4 channels are present in human cardiac c‐kit^+^ progenitor cells. The TRPV2, but not TRPV4, channels regulate cell proliferation. Both TRPV2 and TRPV4 channels participate in regulating cell mobility.

## Conflicts of interest

None.
